# Pregnancy-Associated Venous Thromboembolism: Insights from GARFIELD-VTE

**DOI:** 10.1055/s-0040-1722611

**Published:** 2021-01-27

**Authors:** Carlos Jerjes-Sánchez, David Rodriguez, Alfredo E. Farjat, Gloria Kayani, Peter MacCallum, Renato D. Lopes, Alexander G.G. Turpie, Jeffrey I. Weitz, Sylvia Haas, Walter Ageno, Shinya Goto, Samuel Z. Goldhaber, Pantep Angchaisuksiri, Joern Dalsgaard Nielsen, Sebastian Schellong, Henri Bounameaux, Lorenzo G. Mantovani, Paolo Prandoni, Ajay K. Kakkar, on behalf of the GARFIELD-VTE investigators

**Affiliations:** 1 Tecnologico de Monterrey. Escuela de Medicina y Ciencias de la Salud, Instituto de Cardiologia y Medicina Vascular, TecSalud, Monterrey, Mexico; 2 Thrombosis Research Institute, London, United Kingdom; 3 Queen Mary University of London, London, United Kingdom; 4 Division of Cardiology, Duke University Medical Center, Duke Clinical Research Institute, Durham, North Carolina, United States; 5 McMaster University, Hamilton, Ontario, Canada; 6 Thrombosis and Atherosclerosis Research Institute, Hamilton, Ontario, Canada; 7 Formerly Technical University of Munich, Munich, Germany; 8 Department of Medicine and Surgery, University of Insubria, Varese, Italy; 9 Department of Medicine (Cardiology), Tokai University School of Medicine, Tokai, Japan; 10 Harvard Medical School, Boston, United States; 11 Department of Medicine, Mahidol University, Ramathibodi Hospital, Bangkok, Thailand; 12 Copenhagen University Hospital, Copenhagen, Denmark; 13 Medical Department, Municipal Hospital, Dresden, Germany; 14 Faculty of Medicine, University of Geneva, Geneva, Switzerland; 15 IRCCS Multimedica Milan, Milan, Italy; 16 University of Milano, Bicocca, Milan, Italy; 17 Arianna Foundation on Anticoagulation, Bologna, Italy; 18 University College London, London, United Kingdom

**Keywords:** venous thromboembolism, registry, deep vein thrombosis, pulmonary embolism, pregnancy

## Abstract

**Introduction** The risk of venous thromboembolism (VTE) increases during pregnancy and the puerperium such that VTE is a leading cause of maternal mortality.

**Methods** We describe the clinical characteristics, diagnostic strategies, treatment patterns, and outcomes of women with pregnancy-associated VTE (PA-VTE) enrolled in the Global Anticoagulant Registry in the FIELD (GARFIELD)-VTE. Women of childbearing age (<45 years) were stratified into those with PA-VTE ( *n* = 183), which included pregnant patients and those within the puerperium, and those with nonpregnancy associated VTE (NPA-VTE; *n* = 1,187). Patients with PA-VTE were not stratified based upon the stage of pregnancy or puerperium.

**Results** Women with PA-VTE were younger (30.5 vs. 34.8 years), less likely to have pulmonary embolism (PE) (19.7 vs. 32.3%) and more likely to have left-sided deep vein thrombosis (DVT) (73.9 vs. 54.8%) compared with those with NPA-VTE. The most common risk factors in PA-VTE patients were hospitalization (10.4%), previous surgery (10.4%), and family history of VTE (9.3%). DVT was typically diagnosed by compression ultrasonography (98.7%) and PE by chest computed tomography (75.0%). PA-VTE patients more often received parenteral (43.2 vs. 15.1%) or vitamin K antagonists (VKA) (9.3 vs. 7.6%) therapy alone. NPA-VTE patients more often received a DOAC alone (30.2 vs. 13.7%). The risk (hazard ratio [95% confidence interval]) of all-cause mortality (0.59 [0.18–1.98]), recurrent VTE (0.82 [0.34–1.94]), and major bleeding (1.13 [0.33–3.90]) were comparable between PA-VTE and NPA-VTE patients. Uterine bleeding was the most common complication in both groups.

**Conclusion** VKAs or DOACs are widely used for treatment of PA-VTE despite limited evidence for their use in this population. Rates of clinical outcomes were comparable between groups.

## Introduction

Venous thromboembolism (VTE), which includes deep vein thrombosis (DVT) and pulmonary embolism (PE), is a leading cause of morbidity and mortality worldwide. 1 The risk of VTE is increased fivefold to 10-fold in pregnancy and the puerperium, 2 3 complicating 1 in 1,000 deliveries. 4 VTE is one of the leading causes of maternal mortality in the developed world and causes approximately 1.1 deaths per 100,000 deliveries. 5 The increased risk of VTE in pregnancy and the puerperium reflects, at least in part, the hypercoagulability that has evolved to protect women from hemorrhage at the time of childbirth or miscarriage. It is evident even in the first trimester. 6 The risk of VTE is highest immediately after delivery, specifically for 3 to 6 weeks postpartum, after which the risk declines rapidly. 2 7

Despite the increased risk of VTE, the majority of pregnant women do not require routine thromboprophylaxis, with the exception of those with risk factors, such as previous episode of VTE. 6 8 The diagnosis of pregnancy-associated VTE (PA-VTE) is challenging due to the insidious and nonspecific presentation, in addition to the potential for both fetal and maternal complications and the lack of research in this area. 9

Anticoagulation therapy is the mainstay of prevention and treatment in PA-VTE. Most guidelines recommend low-molecular weight heparin (LMWH) 9 10 11 12 13 ; vitamin K antagonists (VKAs) are not recommended, as they cross the placental barrier and can be teratogenic in the first trimester and associated with an increased risk of fetal hemorrhage in the third trimester. 14 VKAs are, however, sometimes used in the second trimester of pregnancy and early in the third trimester to prevent valve thrombosis in women with mechanical heart valves and could be used in the same manner for VTE treatment. They are also advocated for use during the postpartum period of PA-VTE. 11 Like VKAs, direct oral anticoagulants (DOACs) also cross the placenta. They are contraindicated in pregnancy. Conversely however, DOACs are not recommended for use during the postpartum period. 15 Breastfeeding patients were originally excluded from clinical trials evaluating DOAC safety. Recent studies have shown however, that DOACs are excreted in breastmilk during the lactation stage postpartum. 16 17 Although DOAC dose detection in breast milk was low, the safety for breastfeeding infants has not been adequately determined, largely due to lack of trial participation. 18 How widely these principles are applied in the global management of PA-VTE remains uncertain. The purpose of this analysis was to use data from the Global Anticoagulant Registry in the FIELD (GARFIELD)-VTE to compare patient characteristics and trends in diagnosis and therapy among women with PA-VTE versus nonpregnancy associated VTE (NPA-VTE). PA-VTE included patients who were pregnant at the time of diagnosis, or patients who were within the 6 weeks of puerperium.

## Methods

### Study Design and Participants

The design of GARFIELD-VTE has been described previously. 19 20 Briefly, GARFIELD–VTE (ClinicalTrials.gov identifier: NCT02155491) is an on-going prospective, observational study that enrolled 10,688 VTE patients from 415 sites in 28 countries. The national coordinating investigators identified care settings that most accurately represented the management of VTE patients in their country. Men and women ≥18 years of age with an objectively confirmed diagnosis of VTE within 30 days of entry into the registry were eligible for inclusion. No specific treatments, tests, or procedures are mandated by the protocol. Decisions to initiate, continue, or change treatment were solely at the discretion of the treating physicians and their patients. For this ancillary study, women greater than 45 years of age were excluded from the analysis.

### Data Collection

Data are captured using an electronic case report form (eCRF) designed by eClinicalHealth Services (Stirling, UK) and submitted electronically via a secure website to the registry-coordinating center at the Thrombosis Research Institute, which was responsible for checking the completeness and accuracy of data collected from medical records. The GARFIELD-VTE protocol requires that 10% of all eCRFs are monitored against source documentation, that there is an electronic audit trail for all data modifications, and that critical variables are subjected to additional audit. This study reports data from prospective patients enrolled between the periods May 12, 2014 and January 4, 2017. The data were extracted from the study database on December 8th, 2018.

### Definitions

Women with PA-VTE were defined by the investigators as those of 18 to 45 years of age, diagnosed with VTE during any stage of pregnancy or within 6-weeks postpartum. PA-VTE patients were analyzed collectively and thus were not subcategorized based upon trimester or postpartum stage. The control group of women, those with NPA-VTE, were defined as those with 18 to 45 years of age, diagnosed with a VTE who were neither currently pregnant nor in the 6-week puerperium stage post-pregnancy. Clinical outcomes analyzed were all-cause mortality, recurrent VTE episode, bleeding, and arterial events (myocardial infarction/acute coronary syndromes, stroke/transient ischemic accident) over 12-months from VTE diagnosis. Major bleeding was defined according to the International Society of Thrombosis and Hemostasis criteria. 21 Nonmajor bleeding was defined as any overt bleeding not meeting the criteria for major bleeding. Outcomes were not centrally adjudicated. Active cancer was defined as cancer that was treated ≤90 days before and up to 30 days after VTE diagnosis. History of cancer was defined as a cancer diagnosis >90 days before the diagnosis of VTE, and not currently being treated.

### Ethics Statement

The registry is conducted in accordance with the Declaration of Helsinki and guidelines from the International Conference on Harmonization on Good Clinical Practice and Good Pharmaco-epidemiological Practice and adheres to all applicable national laws and regulations. Independent ethics committee for each participating country and the hospital-based institutional review board approved the design of the registry. All patients provided written informed consent to participate. Confidentiality and anonymity of patients recruited into this registry are maintained.

### Statistical Analysis

These analyses describe data collected at baseline, i.e., within 30 days before or after VTE diagnosis. Continuous variables are presented as mean and standard deviation (SD), and categorical variables are presented as frequency and percentage. Thus, numerical differences are provided only. Patients with missing values were not removed from the study (available case analysis). Percentages are calculated using available data. Hazard ratios were estimated using Cox proportional hazard models adjusted for age, ethnicity, and body mass index (BMI). Statistical analyses were performed using R statistical software and SAS software version 9.4 (SAS Institute Inc., Cary, North Carolina, United States).

## Results

### Baseline Characteristics

This analysis includes a cohort of 1,375 women aged ≤45 years, 13% of the full GARFIELD-VTE registry. Patient recruitment according to country and region showed that the majority of patients were recruited from high-income regions such as Europe and Northern America/Australia ( Appendix Table A1 ). Women with VTE were stratified into those with PA-VTE ( *n* = 183) or NPA-VTE ( *n* = 1187). Baseline characteristics and clinical care pathways are shown in Table 1 .

**Table 1:** Baseline characteristics and care pathways

Variable	NPA-VTE ( *N* = 1,187)	PA-VTE ( *N* = 183)
Age, years, mean (SD)	34.8 (7.0)	30.5 (5.6)
Ethnicity, *n* (%)		
Asian	192 (17.1)	35 (20.1)
Black	129 (11.5)	19 (10.9)
Caucasian	683 (60.7)	95 (54.6)
Other	121 (10.8)	25 (14.4)
Missing	62	9
Smoking status, *n* (%)		
Ex-smoker	100 (8.7)	15 (8.4)
Current smoker	185 (16.1)	2 (1.1)
Missing	37	5
BMI, kg/m ^2^ , mean (SD)	28.2 (8.3)	27.7 (6.4)
Missing	108	12
BMI categories		
< 20	107 (9.9)	12 (7.0)
20–25	361 (33.5)	57 (33.3)
25–30	249 (23.1)	47 (27.5)
30–35	175 (16.2)	37 (21.6)
35–40	95 (8.8)	12 (7.0)
≥40	92 (8.5)	6 (3.5)
Missing	108	12
VTE type, *n* (%)		
DVT	803 (67.6)	147 (80.3)
PE	252 (21.2)	26 (14.2)
DVT and PE	132 (11.1)	10 (5.5)
Site of DVT, *n* (%)		
Upper limb	68 (7.2)	4 (2.5)
Lower limb	847 (90.3)	151 (96.2)
Caval vein	23 (2.4)	2 (1.2)
Missing	249	26
Unilateral or bilateral DVT, *n* (%)		
Left	514 (54.8)	116 (73.9)
Right	383 (40.8)	32 (20.4)
Both	41 (4.4)	9 (5.7)
Missing	249	26
Care setting, *n* (%)		
Hospital	563 (47.4)	111 (60.7)
Outpatient setting	31 (2.6)	3 (1.6)
Specialty, *n* (%)	519 (43.7)	61 (33.3)
Vascular medicine	29 (2.4)	2 (1.1)
General practitioner	45 (3.8)	6 (3.3)
Internal medicine (hematology and intensive care)	563 (47.4)	111 (60.7)
Emergency medicine	31 (2.6)	3 (1.6)
Cardiology	519 (43.7)	61 (33.3)

Abbreviations: BMI, body mass index; DVT, deep vein thrombosis; NPA-VTE, nonpregnancy-associated venous thromboembolism; PA-VTE, pregnancy-associated venous thromboembolism; PE, pulmonary embolism; SD, standard deviation; VTE, venous thromboembolism.

PA-VTE patients were younger than those with NPA-VTE, with a mean age of 30.5 ± 5.6 years and 34.8 ± 7.0 years, respectively ( Table 1 ). Women with both PA-VTE and NPA-VTE were mainly Caucasian (54.6 and 60.7%). A similar proportion in both groups was obese. Women with PA-VTE were less likely to be current smokers (1.1 vs. 16.1%), and more likely to be treated by internal medicine specialists (60.7 vs. 47.4%) than women with NPA-VTE.

PA-VTE patients were less likely to have PE than patients with NPA-VTE (19.7 and 32.3%), and more likely to have DVT of the lower extremities (96.2 and 90.3%, respectively), almost three quarters of which were left-sided in those with PA-VTE (73.2%). In contrast, there was a more equal distribution in women with NPA-VTE (55.0%).

### Risk Factors

Provoking factors must have occurred within the 3 months prior to VTE diagnosis. The most common provoking risk factors in women with PA-VTE were hospitalization (10.4%) and previous surgery (10.4%). Other common predisposing risk factors included a family history of VTE (9.3%) and known thrombophilia (7.7%) ( Table 2 ). In women with NPA-VTE, the most common risk factors were previous surgery (12.0%), previous episode of VTE (10.8%), and oral contraceptive use (34.1%). These women were also more likely to have acute medical illness (6.7 vs. 1.6%), lower limb trauma (8.9 vs. 0.5%), active cancer (4.9 vs. 0.0%), and history of cancer (3.7 vs. 0.5%) than women with PA-VTE.

**Table 2:** Risk factors at baseline

Variable, *n* (%)	NPA-VTE ( *N* = 1,187)	PA-VTE ( *N* = 183)
Provoking risk factors		
Active cancer	58 (4.9)	0 (0.0)
Acute medical illness	79 (6.7)	3 (1.6)
Hospitalization	113 (9.5)	19 (10.4)
Long-haul traveling	66 (5.6)	2 (1.1)
Surgery	142 (12.0)	19 (10.4)
Trauma of the lower limb	106 (8.9)	0 (0.0)
Predisposing risk factors		
Hormone replacement therapy	31 (2.6)	4 (2.2)
Oral contraception	405 (34.1)	4 (2.2)
Recent bleed or anemia	634 (5.3)	9 (4.9)
Chronic heart failure	11 (0.9)	1 (0.5)
Chronic immobilization	51 (4.3)	4 (2.2)
Family history of VTE	95 (8.0)	17 (9.3)
History of cancer	45 (3.8)	1 (0.5)
Known thrombophilia	57 (4.8)	14 (7.7)
Prior episode of DVT and/or PE	128 (10.8)	12 (6.6)
Renal insufficiency	9 (0.8)	0 (0.0)

Abbreviations: DVT, deep vein thrombosis; NPA-VTE, nonpregnancy-associated venous thromboembolism; PA-VTE, pregnancy-associated venous thromboembolism; PE, pulmonary embolism; VTE, venous thromboembolism.

Note: Provoking risk factors occurred during 3 months preceding VTE diagnosis.

### Diagnostic Strategies

DVT was diagnosed by compression ultrasonography in most women with both PA-VTE (98.7%) and NPA-VTE (95.1%). Other imaging tests included computed tomography (1.3 vs. 5.9%) or contrast venography (1.9 vs. 1.7%), respectively. There was infrequent use of pretest clinical probability scoring schemes in both groups (5.2 vs. 1.3%). Measurement of D-dimer levels was reported in a similar proportion of DVT patients with PA-VTE and NPA-VTE (26.8 vs. 25.1%) ( Table 3 ).

**Table 3:** Diagnostic strategies for deep vein thrombosis

Variable, *n* (%)	NPA-DVT ( *N* = 935)	PA-DVT ( *N* = 157)
Confirmatory diagnostic		
Compression ultrasonography	889 (95.1)	155 (98.7)
Vein computed tomography	55 (5.9)	2 (1.3)
Contrast venography	16 (1.7)	3 (1.9)
Impedance plethysmography	2 (0.2)	0 (0.0)
Magnetic resonance angiography	2 (0.2)	0 (0.0)
Other investigation		
D-dimer assay	235 (25.1)	42 (26.8)
Pretest probability scores (e.g., Wells and Hamilton)	49 (5.2)	2 (1.3)

Abbreviations: NPA-VTE, nonpregnancy-associated venous thromboembolism; PA-VTE, pregnancy-associated venous thromboembolism.

Note: Numbers represent the number of tests, not patients. Patients may have received more than one test so values are not mutually exclusive.

The diagnosis of PE was often established with spiral chest CT scan/CT pulmonary angiography in women with both PA-VTE and NPA-VTE (86.1 vs. 92.2%, respectively). Other investigations in women with PE included echocardiography (11.1 vs. 14.1%) and measurement of troponin or B-type natriuretic peptide (BNP) (16.7 vs. 13.8%), respectively ( Table 4 ). Ventilation perfusion scans were used more frequently in women with PA-VTE compared with those with NPA-VTE (22.2 vs. 14.1%).

**Table 4:** Diagnostic strategies for pulmonary embolism

Variable, *n* (%)	NPA-PE ( *N* = 384)	PA-PE ( *N* = 36)
Confirmatory diagnostic		
Any CT	454 (92.2)	31 (86.1)
Ventilation perfusion scan	54 (14.1)	8 (22.2)
Magnetic resonance angiography	0 (0.0)
Other investigation		
Biomarkers (troponin and/or BNP)	53 (13.8)	6 (16.7)
Echocardiography (transthoracic and/or transesophageal)	54 (14.1)	4 (11.1)

Abbreviations: CT, computed tomography; CTPA, computed tomography pulmonary angiography; BNP, B-type natriuretic peptide; NPA-VTE, nonpregnancy-associated venous thromboembolism; PA-VTE, pregnancy-associated venous thromboembolism.

Note: Echocardiography includes transthoracic and/or transesophageal. Any CT includes CTPA, chest CTPA, and spiral CT. Biomarkers include troponin and B-type natriuretic peptide (BNP). Numbers represent the number of tests, not patients. Patients may have received more than one test so values are not mutually exclusive.

### Treatment at Baseline

**Fig. 1:**
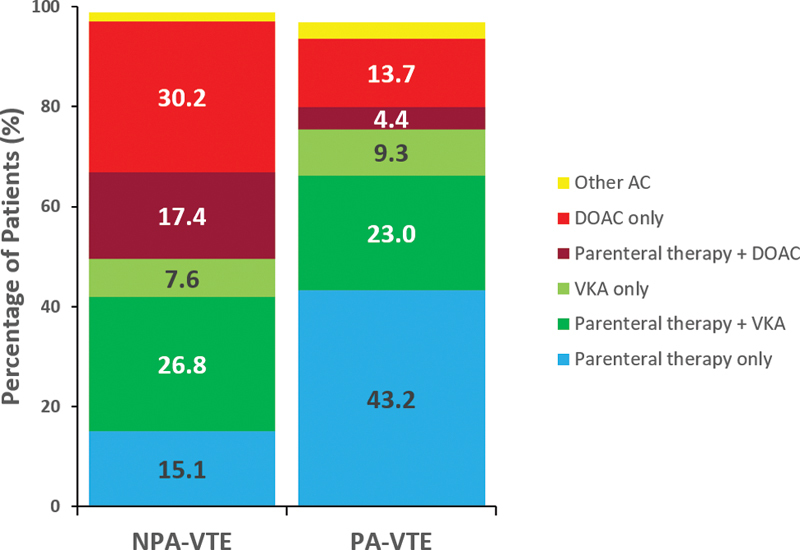
Anticoagulant treatment at baseline (up to 30 days after VTE diagnosis). Percentages are calculated from all patients receiving AC treatment, including those who received AC in combination with other modalities of treatment. AC, anticoagulant; DOAC, direct oral anticoagulant; NPA-VTE, nonpregnancy-associated venous thromboembolism; PA-VTE, pregnancy-associated venous thromboembolism; VKA, vitamin K antagonist.

After VTE diagnosis, most women with PA-VTE (96.7%) and NPA-VTE (98.8%) received anticoagulant therapy ( Fig. 1 ). Women with PA-VTE were more likely to be treated with a parenteral anticoagulant (43.2 vs. 15.1%) or a VKA alone (9.3 vs. 7.6%). Women with NPA-VTE were more likely to be treated with a parenteral anticoagulant in combination with a VKA (26.8 vs. 23.0%) or a DOAC (17.4 vs. 4.4%), or a DOAC alone (30.2 vs. 13.7%) ( Fig. 1 ).

Thrombolytic or fibrinolytic therapy was rarely used both in women with PA-VTE or NPA-VTE (6.0 and 4.2%), as were surgical or mechanical interventions (2.2 and 1.1%, respectively). The distribution of thrombolytic/fibrinolytic therapy among patients with DVT, PE, or both was comparable between the two groups ( Appendix Table A2 ).

### Clinical Outcomes

**Fig. 2:**
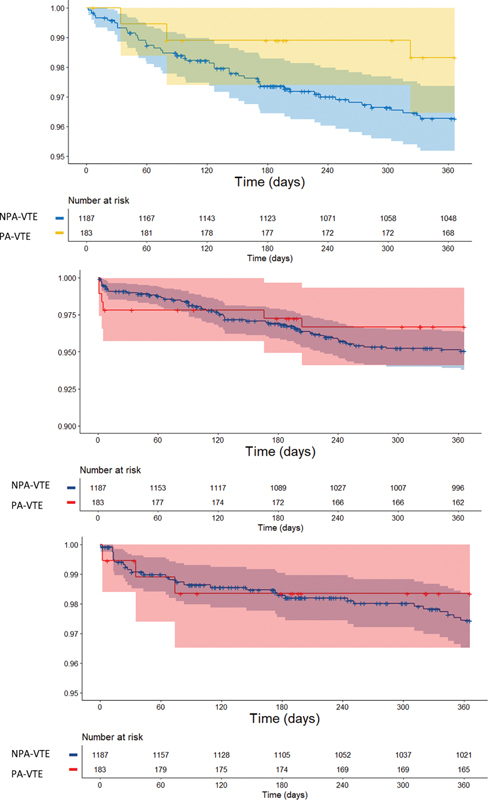
Unadjusted time-to-event curves for ( **A** ) all-cause mortality ( **B** ) recurrent VTE, and ( **C** ) major bleeding. Data are shown as percentage of patients and 95% confidence intervals. NPA-VTE, nonpregnancy-associated venous thromboembolism; PA-VTE, pregnancy-associated venous thromboembolism.

During the 12-months of follow-up, the rates (95% confidence interval) of all-cause mortality were slightly lower in women with PA-VTE than NPA-VTE (1.71 [0.55–5.29] vs. 3.86 [2.87–5.21] per 100-person years, respectively) as were the rates of recurrent VTE, major bleeding, and any bleeding (3.51 [1.58–7.82] vs. 5.19 [4.00–6.75] per 100 person-years, respectively), (1.73 [0.56–5.37] vs. 2.65 [1.84–3.81] per 100 person-years, respectively), and (9.78 [5.99–15.96] vs.13.85 [11.74–16.35] per 100 person-years, respectively). Arterial thrombosis was infrequent in both patient groups ( Table 5 ). The unadjusted time-to-event curves are shown in Fig. 2 .

**Table 5:** Unadjusted event rates (per 100 person-years) 12-mo after VTE diagnosis

	NPA-VTE ( *N* = 1,187)		PA-VTE ( *N* = 183)
	Number of events	Event rate (95% CI)	Number of events	Event rate (95% CI)
All-cause mortality	43	1.71 (0.55–5.29)	3	3.86 (2.87–5.21)
Recurrent VTE	56	3.51 (1.58–7.82)	6	5.19 (4.00–6.75)
Major bleeding	29	1.73 (0.56–5.37)	3	2.65 (1.84–3.81)
Any bleeding	140	9.78 (5.99–15.96)	16	13.85 (11.74–16.35)
Myocardial Infarction/ACS	1	0.57 (0.08–4.05)	1	0.09 (0.01–0.64)
Stroke/TIA	2	N/A	0	0.18 (0.05–0.72)

Abbreviations: ACS, acute coronary syndrome; CI, confidence interval; NPA-VTE, nonpregnancy-associated venous thromboembolism; PA-VTE, pregnancy-associated venous thromboembolism; TIA, transient ischemic attack; VTE, venous thromboembolism.

**Fig. 3:**
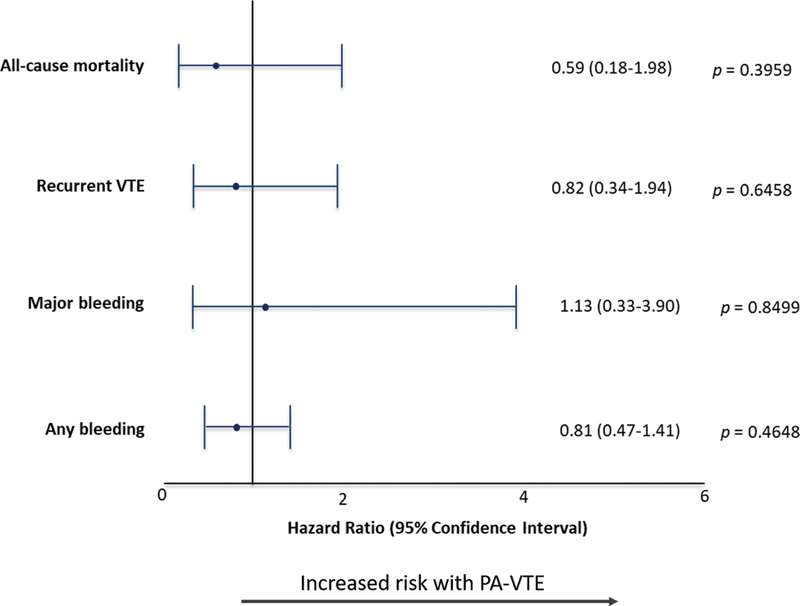
Forest plots for hazard ratios (HRs) with 95% CIs for 12-month outcomes *.* Reference group: nonpregnant women. HRs were adjusted for age, ethnicity, and BMI. BMI, body mass index; PA-VTE, pregnancy-associated venous thromboembolism.

After adjustment for baseline characteristics, the risk of all-cause mortality at 12-months was comparable between women with PA-VTE or NPA-VTE (HR 0.59 [0.18–1.98]) as were the risks of recurrent VTE (HR 0.82 [0.34–1.94]), major bleeding (HR 1.13 [0.33–3.90]), and any bleeding (HR 0.81 [0.47–1.41]) ( Fig. 3 ). Most bleeding events in both groups were uterine (56.3 vs. 55.0%) ( Appendix Table A3 ).

## Discussion

The outcomes of women with PA-VTE were not significantly different from those with NPA-VTE. Women with PA-VTE were younger, more likely to have left-sided DVT, and less likely to have PE. They were more likely to have had hospitalization, a family history of VTE, or thrombophilia. PA-DVT and PE were mostly diagnosed with compression ultrasonography and chest CT, respectively. Around half of all women with PA-VTE received a VKA or a DOAC. PA-VTE patients comprised a low proportion of the overall GARFIELD-VTE cohort.

Women with PA-VTE, at any stage of pregnancy or within 6 weeks of puerperium, were younger than those with NPA-VTE. Just one-fifth of women with PA-VTE had PE (with or without associated DVT), compared with almost one-third of those with NPA-VTE. This finding is in agreement with a previous study 8 that reported PE in only 21% of pregnant women with VTE. This may be attributed to the fact that PE is less frequently searched for in pregnant women as DVT diagnosis is considered sufficient to treat with anticoagulation. As previously reported, left-sided DVT was more common in PA-VTE, 22 likely reflecting exacerbated compression of the left common iliac vein by the right common iliac artery. 23 24

Previous research has identified risk factors for VTE during pregnancy and postpartum. 6 25 26 27 28 29 The Royal College of Obstetricians and Gynaecologists 12 proposed a checklist for risk factors assessment for VTE, including pre-existing risk factors (previous VTE, thrombophilia, obesity), obstetric risk factors (multiple pregnancy, caesarean section), and transient risk factors (hospitalization, immobility). In this analysis, the most common risk factors in women with PA-VTE were hospitalization, previous surgery, family history of VTE, and previous VTE.

Diagnosis of VTE during pregnancy is challenging, as many of the classical signs and symptoms of VTE may also be associated with normal pregnancy. 30 Current recommendations suggest that pregnant women with suspected DVT should have the diagnosis confirmed with compression ultrasonography, 31 reserving magnetic resonance imaging for suspected iliac vein thrombosis. 32 In GARFIELD-VTE, the diagnosis of PE in PA-VTE was primarily established with CT scanning, encompassing spiral CT scanning, and CT pulmonary angiography. Ventilation–perfusion scanning was less common. The D-dimer assay was used in one-quarter of women with PA-VTE. This is in accordance with guidelines from the European Society of Cardiology, 13 which states that normal levels of D-dimer can exclude PE. However, other guidelines, including the Royal College of Obstetricians and Gynaecologists and American Thoracic Society do not recommend the use of D-dimer in pregnancy 33 34 as the levels of this biomarker increase during pregnancy and return to normal approximately 4 to 6 weeks postpartum. In PA-VTE the use of biomarkers and echocardiography was low as observed in recent evidence. 35

Women with PA-VTE were more likely to be initiated on parenteral therapy, typically LMWH, reflecting guideline recommendations. 11 13 34 36 The safety and efficacy of LMWHs for the treatment of VTE in pregnancy have been shown previously, 37 as they do not cross the placenta and are associated with fewer adverse effects than unfractionated heparin. VKA and DOAC usage, with or without co-prescription of parenteral therapy, was high in PA-VTE patients (50.4%). As VKAs are contraindicated for most pregnant patients it is likely that, within PA-VTE patients, VKAs were administered during the puerperium stage. Patients within the PA-VTE group were less likely to receive DOACs, reflecting that they are both contraindicated for pregnant patients and, due to lack of available safety evidence, are also not recommended during the postpartum stage. In particular, DOACs are not recommended for use during the postpartum breastfeeding stage as DOACs are capable of excreting into breastmilk, 16 17 during the postpartum period due to the increased glomerular filtration rate which induces treatment failure, 38 39 and during the postpartum stage immediately post-delivery when the risk of adverse uterine bleeding is at its highest. The design of the registry avoids the identification of the underlying mechanisms of bleeding complications in both groups. However, the primary mechanisms may be related to benign and malignant structural disease and hormonal or functional alterations of the endometrium. 40

It is important to address potential limitations of this study. First, GARFIELD-VTE did not collect information regarding the date of pregnancy in relation to the VTE episode; only that pregnancy was a risk factor. Thus, we are unable to differentiate between antenatal and postpartum patients, nor subcategorize based upon the stage of pregnancy. Data were not collected regarding antenatal risk factors, such as gestational diabetes and multiple pregnancies, or postnatal risk factors, such as caesarean section and preeclampsia, and uterine bleedings. Finally, the sample size of patients with PA-VTE was small, so we may have had insufficient power to detect differences in outcomes.

In conclusion, findings from the GARFIELD-VTE registry demonstrate contemporary demographic characteristics, risk factors, diagnostic, therapeutic trends, and outcomes in PA-VTE patients. VKAs or DOACs are widely used despite limited evidence and uterine bleeding emerges as a relevant outcome.
