# Supplementary material for: Pregnancy-Associated Venous Thromboembolism: Insights from GARFIELD-VTE
Source: TH Open. 2021 Jan 27;5(1):e24–34. doi: 10.1055/s-0040-1722611 (PMC7840428; doi:10.1055/s-0040-1722611)
Supplement: Supplementary file 1 — Supplementary Material [file 10-1055-s-0040-1722611-s200096.pdf]

Appendix

Appendix Table A1 Patient enrolment by country

	NPA-VTE ($n = 1{,}187$)	PA-VTE ($n = 183$)
Country, *n* (%)		
Argentina	29 (2.4)	1 (0.5)
Australia	46 (3.9)	4 (2.2)
Belgium	36 (3.0)	2 (1.1)
Brazil	25 (2.1)	2 (1.1)
Canada	51 (4.3)	5 (2.7)
China	24 (2.0)	13 (7.1)
Czech Republic	79 (6.7)	5 (2.7)
Denmark	14 (1.2)	5 (2.7)
Egypt	124 (10.4)	37 (20.2)
France	62 (5.2)	9 (4.9)
Germany	35 (2.9)	2 (1.1)
Hong Kong	10 (0.8)	0 (0.0)
Italy	45 (3.8)	10 (5.5)
Japan	7 (0.6)	2 (1.1)
Malaysia	44 (3.7)	13 (7.1)
Mexico	10 (0.8)	3 (1.6)
Netherlands	17 (1.4)	3 (1.6)
Russia	86 (7.2)	16 (8.7)
South Africa	117 (9.9)	18 (9.8)
South Korea	15 (1.3)	0 (0.0)
Spain	26 (2.2)	1 (0.5)
Switzerland	11 (0.9)	1 (0.5)
Taiwan	14 (1.2)	1 (0.5)
Thailand	50 (4.2)	3 (1.6)
Turkey	87 (7.3)	16 (8.7)
United Arab Emirates	2 (0.2)	2 (1.1)
United Kingdom	82 (6.9)	8 (4.4)
United States	39 (3.3)	1 (0.5)
Missing	0	0
Region, *n* (%)		
Africa and Middle East	243 (20.5)	57 (31.1)
Asia	164 (13.8)	32 (17.5)
Europe	580 (48.9)	78 (42.6)
Latin America	64 (5.4)	6 (3.3)
North America/Aus	136 (11.5)	10 (5.5)
Missing	0	0

Appendix Table A2 Thrombolytic/fibrinolytic therapy according to VTE type

	NPA-VTE ($n = 1{,}187$)			PA-VTE ($n = 183$)		
	DVT ($n = 803$)	PE ($n = 252$)	DVT + PE ($n = 132$)	DVT ($n = 147$)	PE ($n = 26$)	DVT + PE ($n = 10$)
Thrombolytic/Fibrinolytic therapy, *n* (%)						
No	778 (96.9)	241 (95.6)	118 (89.4)	139 (94.6)	25 (96.2)	8 (80.0)
Yes	25 (3.1)	11 (4.4)	14 (10.6)	8 (5.4)	1 (3.8)	2 (20.0)
Missing	0	0	0	0	0	0

Abbreviations: DVT, deep vein thrombosis; NPA, nonpregnancy associated VTE; PA-VTE, pregnancy-associated VTE; PE, pulmonary embolism; VTE, venous thromboembolism.

 © 2021. The Author(s).

Appendix Table A3 Site of bleeding (major and minor)

Site of bleeding, *n* (%)	NPA-VTE (*N* = 140)	PA-VTE (*N* = 16)
Uterine	77 (55.0)	9 (56.3)
Epistaxis	14 (10.0)	2 (12.5)
GI lower	8 (5.7)	1 (6.2)
Intramuscular (no compartment syndrome)	3 (2.1)	1 (6.2)
Puncture site	1 (0.7)	1 (6.2)
Skin (ecchymosis other than instrument site)	5 (3.6)	1 (6.2)
Macroscopic hematuria	2 (1.4)	0 (0.0)
Gingival	9 (6.4)	0 (0.0)
Intraperitoneal	2 (1.4)	0 (0.0)
Hemoptysis	5 (3.6)	0 (0.0)
Hemopericardium	1 (0.7)	0 (0.0)
Hemothorax	1 (0.7)	0 (0.0)
Other	11 (7.9)	1 (6.2)
Unknown	1 (0.7)	0 (0.0)

 © 2021. The Author(s).

Supplementary Material

Ab Loualidi, Abdurrahim Colak, Abraham Bezuidenhout, Abu Abdool-Carrim, Addala Azeddine, Adriaan Beyers, Adriaan Dees, Ahmed Mohamed, Ahmet Aksoy, Akihiko Abiko, Akinori Watanabe, Alan Krichell, Alberto Alfredo Fernandez, Alberto Tosetto, Alexey Khotuntsov, Alisha Oropallo, Alison Slocombe, Allan Kelly, Amanda Clark, Amr Gad, Amy Arouni, Andor Schmidt, Andrea Berni, Andres Javier Kleiban, Andrew Machowski, Andrey Kazakov, Angel Galvez, Ann Lockman, Anna Falanga, Anoop Chauhan, Antoni Riera-Mestre, Antonino Mazzone, Armando D'Angelo, Artur Herdy, Atsushi Kato, Ayman Abd Elhamid Ebrahim Mahmoud Salem, Azlan Husin, Barbara Erdelyi, Barry Jacobson, Beatrice Amann-Vesti, Bektas Battaloglu, Benedicte Wilson, Benilde Cosmi, Bergmann Jean Francois, Berremeli Toufek, Beverley Hunt, Bhavesh Natha, Bisher Mustafa, Bonnie Chi Shan Kho, Boulon Carine, Brian Zidel, Brisot Dominique, Brousse Christophe, Bruno Trimarco, Canhua Luo, Carlos Alberto Cuneo, Carlos Jerjes Sanchez Diaz, Carsten Schwencke, Cas Cader, Celal Yavuz, Cesar Javier Zaidman, Charles Lunn, Chau-Chung Wu, Cheng Hock Toh, Chern-En Chiang, Chevrier Elisa, Chien-Hsun Hsia, Chien-Lung Huang, Chi-Hang Kevin Kwok, Chih-Cheng Wu, Chi-Hung Huang, Chris Ward, Christian Opitz, Christina Jeanneret-Gris, Chung Yin Ha, Chun-Yao Huang, Claude Luyeye Bidi, Clifford Smith, Cornelia Brauer, Corrado Lodigiani, Couturaud Francis, Cynthia Wu, Daniel Staub, Daniel Theodoro, Daniela Poli, David—Riesco Acevedo, David Adler, David Jimenez, David Keeling, David Scott, Davide Imberti, Desmond Creagh, Desmurs-Clavel Helene, Dirk Hagemann, Dirk Le Roux, Dirk Skowasch, Dmitry Belenky, Dmitry Dorokhov, Dmitry Petrov, Dmitry Zateyshchikov, Domenico Prisco, Dorthe Møller, Dusan Kucera, Ehab M. Esheiba, Elizaveta Panchenko, Elkouri Dominique, Emre Dogan, Emre Kubat, Enrique Diaz Diaz, Eric Wai Choi Tse, Erik Yeo, Erman Hashas, Ernst Grochenig, Eros Tiraferri, Erwin Blessing, Escande Orthlieb Michèle, Esther Usandizaga, Ettore Porreca, Fabian Ferroni, Falvo Nicolas, Félix Ayala-Paredes, Firas Koura, Fitjerald Henry, Franco Cosmi, Frans Erdkamp, Gadel Kamalov, Garcia-Bragado Dalmau, Garrigues Damien, Garry Klein, Gaurand Shah, Geert Hollanders, Geno Merli, Georg Plassmann, George Platt, Germain Poirier, German Sokurenko, Ghassan Haddad, Gholam Ali, Giancarlo Agnelli, Gin Gin Gan, Grace Kaye-Eddie, Gregoire Le Gal, Gregory Allen, Guillermo Antonio Llamas Esperón, Guillot Jean-Paul, Hagen Gerofke, Hallah Elali, Hana Burianova, Hans-Juergen Ohler, Haofu Wang, Harald Darius, Harinder S. Gogia, Harry Striekwold, Harry Gibbs, Hatice Hasanoglu, Hatice Turker, Hendrik Franow, Henri Bounameaux, Herbert De Raedt, Herman Schroe, Hesham Salah ElDin, Hesham Zidan, Hiroaki Nakamura, Ho Young Kim, Holger Lawall, Hong Zhu, Hongyan Tian, Ho-Young Yhim, Hugo ten Cate, Hun Gyu Hwang, Hyeok Shim, Igor Kim, Igor Libov, Igor Sonkin, Igor Suchkov, Ik-Chan Song, Ilker Kiris, Ilya Staroverov, Irene Looi, Isabel M De La Azuela Tenorio, Ismail Savas, Ivan Gordeev, Ivo Podpera, Jae Hoon Lee, Jameela Sathar, James Welker, Jan Beyer-Westendorf, Jan Kvasnicka, Jan Vanwelden, JangYong Kim, Jaromira Svobodova, Jaspal Gujral, Javier Marino, Javier Tristan Galvar, Jeannine Kassis, Jen-Yuan Kuo, Jhih-Yuan Shih, Ji Hyun Kwon, Jin Hyun Joh, Jin Hyun Park, Jin Seok Kim, Jinghua Yang, Jiri Krupicka, Jiri Lastuvka, Jiri Pumprla, Jiri Vesely, Joan Carlos Souto, João Antônio Correa, Johan Duchateau, John Perry Fletcher, Jorge del Toro, Jorge del Toro, Jorge Guillermo Chavez Paez, Jørn Nielsen, Jose Dalmo Araujo Filho, Jose Saraiva, Jose Antonio Diaz Peromingo, Jose Gomez Lara, Jose Luis Fedele, Jose Maria Surinach, Joseph Chacko, Juan Antonio Muntaner, Juan Carlos Álvarez Benitez, Juan Moreno Hoyos Abril, Julian Humphrey, Julio Bono, Junji Kanda, Juree Boondumrongsagoon, Kai Hang Yiu, Kanchana Chansung, Karin Boomars, Kate Burbury, Katsuhiro Kondo, Kemal Karaarslan, Kensuke Takeuchi, Knut Kroeger, Konstantin Zrazhevskiy, Koscál Svatopluk, Kou-Gi Shyu, Kristel Vandenbosch, Kuan-Cheng Chang, Kuan-Ming Chiu, Kubina Jean-Manuel, Kwan Jing Wern, Kwo-Chang Ueng, Lalita Norasetthada, Laure Binet, Lee Ping Chew, Lei Zhang, Leone Maria Cristina, Lidwine Tick, Lilia Beatriz Schiavi, Lily Lee Lee Wong, Lohana Borges, Louis Botha, Luc Capiau, Luc Timmermans, Luciano Eduardo López, Luigi Ria, Luis Manuel hernandez Blasco, Luis Alberto Guzman, Luis Flota Cervera, Mahe Isabelle, Manuel Monreal Bosch, Manuel de los Rios Ibarra, Manuel Núñez Fernandez, Marc Carrier, Marcelo Raul Barrionuevo, Marco Antonio Alcocer Gamba, Marco Cattaneo, Marco Moia, Margaret Bowers, Mariam Chetanachan, Mario Alberto Berli, Mark Fixley, Markus Faghih, Markus Stuecker, Marlin Schul, Martin Bandai, Martin Koretzky, Martin Myriam, Mary Elizabeth Gaffney, Masao Hirano, Masashi Kanemoto, Mashio Nakamura, Mersel Tahar, Messas Emmanuel, Michael Kovacs, Michael Leahy, Michael Levy, Michael Munch, Michael Olsen, Michel De Pauw, Michel Gustin, Michiel Van Betsbrugge, Mikhail Boyarkin, Miroslav Homza, Modise Koto, Mohamed Abdool-Gaffar, Mohamed Ayman Fakhry Nagib, Mohamed El-Dessoki, Mohamed Khan, Monniaty Mohamed, Moo Hyun Kim, Moon-Hee Lee, Mosaad Soliman, Mostafa Shawky Ahmed, Mostafa Soliman Abd el Bary, Moustafa A. Moustafa, Muhammad Hameed, Muhip Kanko, Mujibur Majumder, Nadezhda Zubareva, Nicola Mumoli, Nik Azim Nik Abdullah, Nisa Makruasi, Nishen Paruk, Nonglak Kanitsap, Norberto Duda, Nordiana Nordin, Ole Nyvad, Olga Barbarash, Orcun Gurbuz, Oscar Gomez Vilamajo, Oscar Nandayapa Flores, Ozcan Gur, Oztekin Oto, Pablo Javier Marchena, Pantep Angchaisuksiri, Patrick Carroll, Pavel Lang, Peter MacCallum, Peter Baron von Bilderling, Peter Blombery, Peter Verhamme, Petr Jansky, Peuch Bernadette, Philippe De Vleeschauwer, Philippe Hainaut, Piera Maria Ferrini, Piriyaporn Iamsai, Ponchaux Christian, Pongtep Viboonjuntra, Ponlapat Rojnuckarin, Prahlad Ho, Pramook Mutirangura, Rachel Wells, Rafael Martinez, Raimundo Tirado Miranda, Ralf Kroening, Rapule Ratsela, Raquel Lopez Reyes, Raul Franco Diaz de Leon, Raymond Siu Ming Wong, Raz Alikhan, Reinhold Jerwan-Keim, Remedios Otero, Renate Murena-Schmidt, Reto Canevascini, Richard Ferkl, Richard White, Rika Van Herreweghe, Rita Santoro, Robert Klamroth, Robert Mendes, Robert Prosecky, Roberto Cappelli, Rudolf Spacek, Rupesh Singh, Sam Griffin, Sang Hoon Na,

 © 2021. The Author(s).

Sanjeev Chunilal, Saskia Middeldorp, Satoshi Nakazawa, Sebastian Schellong, See Guan Toh, Seinturier Christophe, Selim Isbir, Selma Raymundo, Seng Kiat Ting, Serge Motte, Serir Ozkan Aktogu, Servaas Donders, Seung Ick Cha, Seung-Hyun Nam, Sevestre-Pietri Marie-Antoinette, Shaun Maasdorp, Shenghua Sun, Shenming Wang, Sherif Mohamed Essameldin, Sherif Mohamed Sholkamy, Shintaro Kuki, Shinya Goto, Shuichi Yoshida, Shunzo Matsuoka, Simon McRae, Simon Watt, Siriwimon Patanasing, Siwe-Nana Jean-Léopold, Somchai Wongkhantee, Soo-Mee Bang, Sophie Testa, Stanislav Zemek, Steffen Behrens, Stephan Dominique, Stuart Mellor, Suaran Singh Gurcharan Singh, Sudip Datta, Sunee Chayangsu, Susan Solymoss, Tamara Everington, Tarek Ahmed Adel Abdel-Azim, Tawatchai Suwanban, Taylan Adademir, Terence Hart, Terriat Béatrice, Thifhelimbilu Luvhengo, Thomas Horacek, Thomas Zeller, Tim Boussy, Tim Reynolds, Tina Biss, Ting-Hsing Chao, Tomas Smith Casabella, Tomoya Onodera, Tontanai Numbenjapon, Victor Gerdes, Vladimir Cech, Vladimir Krasavin, Vladimir Tolstikhin, W.A. Bax, Wagih Fawzy Abdel Malek, Wai Khoon Ho, Walter Ageno, Walter Pharr, Weihong Jiang, Wei-Hsiang Lin, Weihua Zhang, Wei-Kung Tseng, Wen-Ter Lai, Wilfried De Backer, Wilhelm Haverkamp, Winston Yoshida, Wolfgang Korte, Won Il Choi, Yang-K$_i$ Kim, Yasuhiro Tanabe, Yasushi Ohnuma, Yeung-Chul Mun, Yohan Balthazar, Yong Park, Yoshisato Shibata, Yuriy Burov, Yuriy Subbotin, Zdenek Coufal, Zhenwen Yang, Zhicheng Jing, Zhicheng Jing, Zhongqi Yang.

 © 2021. The Author(s).
